# Analysis of Camel Milk Market Chain in Borana and West Guji Zones, Oromia Regional State, Ethiopia

**DOI:** 10.1002/fsn3.71127

**Published:** 2025-10-25

**Authors:** Hussen Abduku, Mitiku Eshetu, Takele Wolkaro, Tesfemariam Berhe, Temesgen Jembere

**Affiliations:** ^1^ Department of Animal Science Bule Hora University Bule Hora Ethiopia; ^2^ Haramaya University, School of Animal and Range Science Haramaya Ethiopia; ^3^ Bio and Emerging Technology institute (BETin) Addis Ababa Ethiopia; ^4^ Ethiopian Institute of Agriculture Research Addis Ababa Ethiopia

**Keywords:** camel milk, challenge, market chain, milk marketing, opportunities

## Abstract

In arid and semiarid areas, camel milk plays a significant role in improving the livelihoods of the pastoralists. Nowadays, the interest in camel milk production and marketing has been growing, associated with increased demand, rapid urbanization, and livelihood diversification. Despite the production potential, in Ethiopia, the camel milk dairy sector does not receive sufficient attention in the national research and development agenda. This study aimed to analyze the camel milk market chain in selected districts of the Borana and West Guji zones, southern Ethiopia. Using a multistage sampling procedure, the study selected 235 producers, 107 consumers, 92 traders, and 47 transporters. Results revealed that the average camel ownership per household was 16.7 ± 5.88, with average daily milk yields of 5.92 ± 0.89 L during the wet season and 4.72 ± 0.83 L during the dry season. On average, households produced 11.95 L of milk per day; out of production, 29.6% was consumed and 62.95% was sold. Four distinct milk market channels were identified. Producers, traders, retailers, consumers, transporters, and service providers were the key market chain actors. The regression model analysis result indicated that family size, milk production and consumption levels, religion, educational status, milk price, and access to market information significantly influenced the volume of milk supplied to the market (*p* < 0.01). Feed and water scarcity, disease, inadequate market infrastructure, and insufficient extension services were the key production challenges identified. To enhance production and market profitability, governmental and nongovernmental organizations should focus on improving feed and water, developing market infrastructure, facilitating market linkages, and expanding veterinary services.

## Graphical abstract

Out of the total production, 29.6% and 62.95% of milk were allocated for consumption and market supply, respectively. Among the four market channels identified, producers to traders to consumers account for 45.9% of marketed milk. Producers, traders, retailers, consumers, transporters, and service providers were the key market chain actors. Family size, amount of milk produced, religion, amount of milk consumed, education status, milk price, and market information access significantly determine the amount of milk market supply.

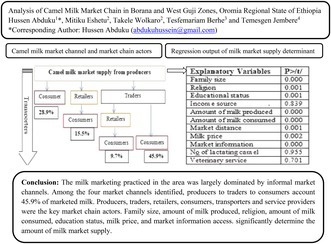

## 1 Introduction

The world's camel population is approximately 40.75 million, of which the one‐humped camel ( *Camelus dromedarius* ) accounts for 95%, and out of this, the majority (> 80%) are inhabited in Africa (Food and Agriculture Organization (FAO) 2020). Ethiopia, Kenya, Sudan, and Somalia account for the largest (42.7%) share. According to the CSA (Central Statistical Agency) 2022, Ethiopia holds 7 million heads, which accounts for around 17.17% of the world population. Camels are uniquely adapted and produce more milk than other dairy species, and they are a reliable source of milk and milk products in arid and semi‐arid areas. Kenya is the leading camel milk producer in the world, followed by Somalia, Pakistan, and Ethiopia (FAO 2022).

In Ethiopia, the pastoral communities rear camels for different purposes, such as sources of food, transportation, and social and cultural purposes (Bekele et al. 2002, 2021). Camel dairy could not only provide more food for the people but also serve as an additional income source. The camel milk contributes more than 50% of the nutrient intake in the pastoral areas (Oromia Pastoral area Development Coordination Commission (OPaDCC) 2021; Mahdi et al. 2025). In Ethiopia, for the last decade from 2011 to 2020, average annual milk production increased by 7.23% (FAO 2022). Based on the milk source, out of the total amount of milk produced annually, camel milk accounts for 25% (1.4 billion liters), next to cow milk (CSA (Central Statistical Agency) 2022).

Studies done so far indicate camel milk production has gained global attention and market exposure (Ait El Alia et al. 2025). In addition to the growing demand for camel milk associated with the rapid urbanization in the eastern part of Ethiopia, camel milk producers reported that the market demand has been increasing (Wako et al. 2022). The small towns and urban areas get their daily milk from their nearby milk producers (Kena 2022). The economic diversification of the pastoralists creates an opportunity for camel producers (Farnworth et al. 2018).

In Ethiopia, around 60% of the total landmass is classified as arid or semiarid (Megersa et al. 2024), where camel keeping is among the most efficient forms of land use. Likewise, the OPaDCC (2021) reported that Oromia pastoral areas cover 152,070 km^2^ (43% of the land mass) and constitute 100% of the camel resource. Recently, camel herding is expanding among Ethiopia's traditionally cattle‐herding pastoralists, such as in Borana and west Guji zones (OPaDCC 2021). This expansion is driven by the gradual alteration of vegetation cover from grass to browse species due to climate change (Tadesse et al. 2013). This increasing trend of camel herding was also reported in Sub‐Saharan African countries, including Northern Kenya and Central Kenya, and the Sahel (Megersa et al. 2024; Wako et al. 2022).

Despite the standing of Ethiopia in Africa in terms of camel population and milk production potential, the country has been neglected in research and development initiatives and also lags in milk and milk product processing and marketing (Megersa et al. 2024; OPaDCC 2021). Additionally, the camel milk marketing and processing industry currently faces critical barriers related to hygiene and limited government support, which hinder quality control and market growth (Ait El Alia et al. 2025). A recent study finding also indicates that the lack of a well‐organized milk market, poor market infrastructure, and market inaccessibility were the challenges that hindered milk marketing (Mahamud 2022; Megersa et al. 2024; Bekele et al. 2021). Therefore, assessing the production potential, milk marketing, as well as the current status of market chain actors is important.

Furthermore, so far, many scholars have investigated the camel milk marketing and handling done in eastern Ethiopia (Legesse et al. 2023; Gebremichael et al. 2019; Wolkaro et al. 2017). However, information about the status of camel milk production potential, milk marketing, and challenges hindering their effectiveness across the market chain in Borana and West Guji zones was limited (Bekele et al. 2021). Thus, this study was paramount to fill the existing information gap and develop insights about appropriate camel dairy development interventions that would lay the groundwork for producers, traders, and policymakers. A particular analysis of the camel milk market chain and assessment of factors affecting the amount of milk supplied to the market in selected districts of Borena and West Guji Zones was the aim of this study.

## 2 Materials and Methods

### 2.1 Study Area

**FIGURE 1:**
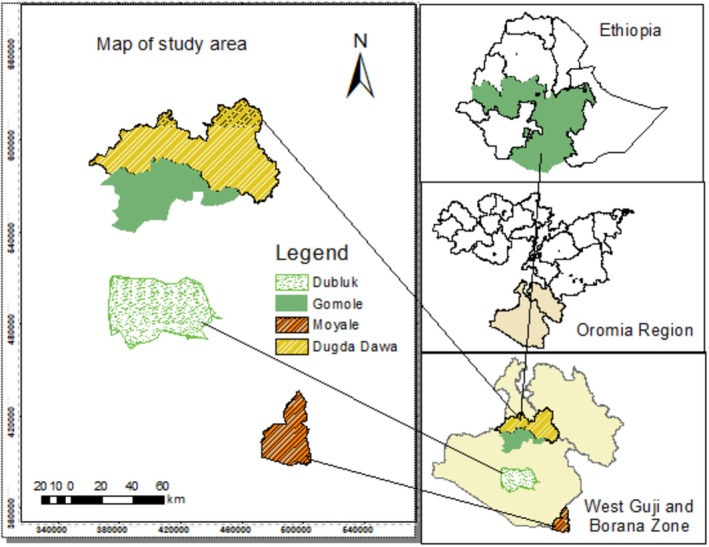
The map of the study area.

The study was carried out in the Oromia region, Dugda Dawa, Gomole, Dubluk, and Moyale districts, Southern Ethiopia. The study area map is shown in Figure 1. Dugda Dawa is 500 km from Addis Ababa and 30 km from the West Guji Zone Bule Hora town. The district had 16 kebeles (smallest administrative unit in Ethiopian context); land cover 3864 km^2^, located at 50°24′4″N latitude and 38°16′24″E longitude. The altitude ranges from 1100 to 1650 m above sea level (m.a.s.l.). Around 70% of the district is lowland, and 30% is mid‐altitude. The annual temperature ranges from 16°C to 30°C (Dugda Dawa District, Agricultural and Natural Resource Office (DuDAgNRo) (2023)). The Gomole district is found 570 km from Addis Ababa and 42 km from the zonal town Yabello. Altitude ranges from 900 to 1650 m.a.s.l. geographically located at 5°8′24″ N, 38°17′51″E, it covers 1286.99 km^2^ of land. The mean annual temperature ranges from 17°C to 30°C (Gomole District, Agricultural and Natural Resource Office (GDAgNRo) (2023)).

The Dubluk district is found at a distance of 640 km from Addis Ababa. According to the Dubluk District, Agricultural and Natural Resource Office (DDAgNRo) 2023, geographically, the district is located at 4°22′0″N, 38°18′0″E; the altitude ranges from 1100 to 1500 m.a.s.l. The Moyale district is found 770 km from Addis Ababa, geographically located at 3°33′43″ N and 39°2′42″ E, bordered by Kenya, Dire, and the Somali region from the south, west, and east, respectively. The altitude ranges from 900 to 1347 m.a.s.l. The area of land cover is 1481 km^2^ (Moyale District Agriculture and Natural Resource office (MDAgNo) 2023). Annual rainfall ranges from 500 to 600 mm with a mean temperature of 37°C (Legesse et al. 2023).

The agroclimatic condition of the area is characterized as semiarid and arid; the vegetation cover is dominated by savannah with mixtures of perennial herbaceous and woody plants such as *Cactus*, *Acacia*, *Cenchrus ciliaris* , *Themeda triandra* , *Chloris roxburghiana*, and thorny shrubs (OPaDCC 2021). The rainfall condition of the area is bimodal and erratic ranging from 400 to 700 mm; it receives 50%–60% in the long rainy season (*Gena*) from mid‐March to May and 25%–30% during the short rainy season (*Haggaya*), which starts in mid‐September and ends around mid‐November. In the area, livestock and livestock product marketing were the major sources of household income (Birru et al. 2017). Rain‐fed agriculture such as maize, wheat, barley, pulses, teff, and haricot bean, was a minor income source (OPaDCC 2021).

### 2.2 Study Design and Framework of the Study

The general methodological framework of the study followed a Value Chain Analysis Approach as described by Achchuthan and Kajananthan (2012); however, due to the scope of the study, its application had been particularly limited to analyzing only the market chain aspects, which include milk production, market channels, price dynamics, and involvement of actors across the market chain. A cross‐sectional study design was adopted to investigate the camel milk market chain and to identify the determining factor for the amount of milk market supply in the study area. A pilot survey was conducted to refine survey questions and determine how appropriately they address the different actors. The actual survey work was conducted from March to September 2024 using semistructured questionnaires, focus group discussions, key informant interviews, and personal observations. To guide group discussions, a concise checklist was prepared, as recommended by FAO (2020). The study population includes milk producers, traders, retailers, transporters, and consumers found in the study area, while the local government administrative offices at zonal district and kebele levels, including agricultural and natural resources offices, cooperative promotion offices, and trade and development offices, were all involved in providing the data required for the study.

#### 2.2.1 Sample Size and Sampling Procedure

To select respondent households, a multistage sampling technique was used. In the first stage, out of six Oromia pastoral zones, the Borana and West Guji zones were purposively selected based on the camel population, milk production potential, and milk market contribution. In the second stage, Dugda‐Dawa district was selected from West Guji, while Gomole, Dubluk, and Moyale districts were selected from the Borana zone purposively based on the camel population, milk production potential, and market access. In the third stage, a purposive sampling procedure was applied to select 12 rural *kebeles*, with three kebeles chosen from each district. Specifically, Chame, Jigesa, and Biyo‐Kuni were selected from the Dugda‐Dawa district; Haro‐Bake, Surupha‐Badiya, and Dadacha‐Kufa from the Gomole district; Kersa‐Danbi, Fulo‐Bika, and Bokossa from the Dubluk district; and Laga‐Sure, Bokkola, and Arable were selected from the Moyale district. The selection of sampled rural *kebeles* was based on the camel population, milk production potential, and accessibility to the market center for milk sales.

Finally, individual respondent HH was sampled after a discussion with the development agent and *Keble* administrators. First, from a total of camel‐rearing households registered in each selected *kebele*, only those pastoralists owning at least two lactating camels were selected. Next, from those listed households, individual respondents were selected using a systematic random sampling technique, ensuring that selected sample households were spread evenly across the sampling frame. In this regard, the total sample size required for the study (235), as indicated in Table 1, was determined based on a formula developed by Kothari (2004), which is expressed as follows:

(1) $$n=\frac{Z^{2}\times N\times p\times q}{{\left(e\right)}^{2}\left(N-1\right)+Z^{2}pXq}$$

where *n* = the sample size, *N* = number of milk producer households (12,669), *Z* = coefficient of normalization (1.96), *q* = probability of failure (0.5), *p* = proportion of the population (0.31), and e = marginal error: 0.05 with 95% CI. The proportional probability sampling approach was used to distribute the households across the rural kebele using Equation (2).

(2) $$\mathrm{ni}=\frac{\mathrm{Ni}\times n}{N}$$

where ni = sample size ratio to be estimated, Ni = camel milk producer population proportion, *n* = the sample size, and *N* = the total population.

**TABLE 1:** Sample size and sampling frame.

Study area (District)	Zone	Producers households		Market chain actors			
				Trader	Consumer	Transporter	Total
		N	%				
Dugda Dawa	West Guji	51	231.8	17	20	9	46
Gomole	Borana	80	34	27	31	14	72
Dubluk	Borana	56	23.8	22	26	11	59
Moyale	Borana	48	20.4	26	30	12	68
Total		235	100	92	107	47	245

*Source:* Own survey data, 2025.

Before data collection, an in‐depth literature review and key informant interviews were conducted to characterize the role and participation of camel milk traders, transporters, and consumers along the market chain, and determine the number of respondents from each group. As shown in Table 1, a total of 92 traders, 47 transporters, and 107 consumers were selected from across the market chain based on Collin (1986) as indicated in Equation (3) below. The size of the respondents selected from each group was determined using a proportional probability to size approach based on milk market share and milk consumption. An individual respondent was selected using the systematic random likelihood sampling technique.

(3) $$n=\frac{t^{2}\times p\times q}{{\left(e\right)}^{2}}$$

where *n* = sample size, *t* = significance level (95%), *p* = probability of the situation being searched (probability of household consuming milk, trading, or transporting in percentage), *q* = the probability of HH not consuming/trading camel milk (1−p), and e = the accepted error (5%).

#### 2.2.2 Conceptual Framework of the Study

**FIGURE 2:**
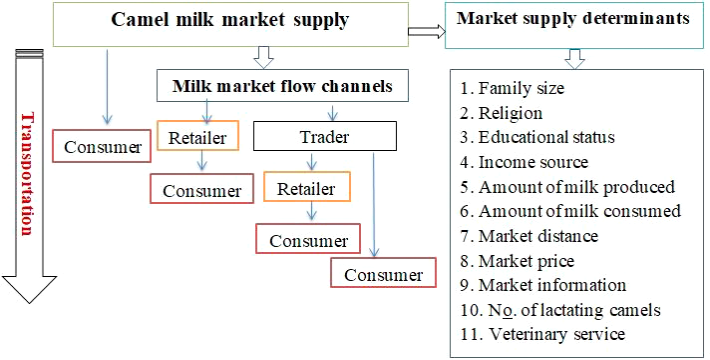
Schematic presentation of the conceptual Framework of the study. (Arrows indicate milk flow along the market channel)

The conceptual framework used to analyze the camel milk market chain and identify the specific milk flow channels across the study area is indicated in Figure 2.

### 2.3 Data Type and Source

In this study, both primary and secondary data sources were utilized. Primary data included the socioeconomic characteristics of respondent households, such as gender, family size, educational status, and religion; herd composition; camel holding size; lactation length; calving interval; milk yield; and the amount of milk consumed and sold. Additional primary data covered milk prices, transportation, input supply, market outlets, and service providers. Secondary data were obtained from local governmental offices, NGOs, and relevant literature. A semi‐structured questionnaire was developed separately for each group to collect the quantitative and qualitative data. First, the questionnaire was prepared in the English language, and then translated into *Afan Oromo* for ease of understanding and management. Four enumerators, one for each district, were also trained on the data collection section of the methodology and specific variables of the research interest. The questionnaire was pre‐tested in each case using 5% non‐randomly selected representatives based on FAO's recommendation to check for any inconsistencies and wording errors.

To gain insights into camel milk marketing, challenges, and opportunities, and triangulate primary data, four Focus Group Discussions (FGD) were conducted, one per district. A total of 34 participants, male (76.47%) and female (23.5%), took part, including members from the kebele administration and elder community members. The number of participants in each group ranged from 8 to 10, with respondents selected based on their experience and willingness to share their opinions. In addition, six Key Informant Interviews (KIIs) were conducted in each study district. These interviews were carried out to clarify the data collection strategy and to determine the number of producers, traders, and transporters sampled across the milk market chain. Informants were selected based on their roles and expertise from district agriculture, trade, and cooperative offices.

### 2.4 Statistical Data Analysis

#### 2.4.1 Descriptive Statistics

Descriptive statistics like mean, percentage, and frequency were used. The data was coded, entered into Microsoft Excel, and analyzed using the Statistical Package for the Social Sciences (SPSS) version 25. To analyze the mean difference of variables, including holding size, calving interval, lactation length, milk market price, and the amount of milk produced, consumed, and sold across the district, the analysis of variance was used. While categorical variables were analyzed using chi‐square (*x* ^2^) and significant differences were declared at *p* < 0.05. The milk market flow channel was developed using survey data collected, and the challenges of milk producers and traders were analyzed using the index ranking method as described by Kosgey (2004). Index = Sum of (5X rank 1 + 4X rank 2 + 3X rank 3 + 4X rank 4 + 1X rank 5) given for an individual reason divided by the sum of (5X rank 1 + 4X rank 2 + 3X rank 3 + 4X rank 4 + 1X rank 5) for overall reasons.

#### 2.4.2 Inferential Analysis

To identify the determining factors that affect the milk market supply, a simple linear regression model was used as described by Gujarati (2003). The regression coefficient (β) value indicates the direction of the relationship; a positive value shows that when one variable increases, the other also increases, while a negative value shows an inverse relationship. To explain the degree of variation due to selected variables, the coefficient of variation was estimated using the adjusted *R*‐squared value.

##### 2.4.2.1 Variable Selection and Model Hypothesis

Total family size, religion, educational status, other source of household income, amount of milk produced in liters, amount of milk consumed in liters, market distance in kilometers, milk market price, market information access, number of lactating camels, and access to veterinary services were the explanatory variables selected; they were selected based on the previous study findings or research gaps and relevance to the milk marketing.

##### 2.4.2.2 Model Specification

As recommended by Gujarati (2003), the multiple regression models were used to identify the determining factors and to predict the value of the dependent variable based on the value of the independent variables. The following regression equation was used:

$$\begin{array}{cc} \mathrm{Yi}= & \text{β0}+\beta 1\text{X1}+\beta 2\text{X2}+\beta 3\text{X3}\\ & +\beta 4\text{X4}+\beta 5\text{X5}+\beta 6\text{X6}+\beta 7\text{X7}+\beta 8\text{X8}\\ & +\beta 9\text{X9}+\beta 1\text{0X}10+\beta 1\text{1X}11+\mathrm{\varepsilon i} \end{array}$$

where Yi refers to the amount of milk supplied for market (L).

β₀ = constant term, Independent variables were total family size (X1), religion (X2), educational status (X3), other source of household income (X4), amount of milk produced (X5), amount of milk consumed (X6), market distance (X7), milk market price (X8), market information access (X9), number of lactating camels (X10), and access to veterinary service (X11). β1, β2, β3, β4, β5, β6, β7, β8, β9, β10, and β11 are coefficients of each respective independent variable. εi = the residual variance in Y after taking into consideration the effects of the Xi variables included in the model. Mathematically, Y and X have a linear association in which any change in each of the Xi results in a change in Y and vice versa.

Before fitting the variables into a model, the post‐estimation diagnostic analysis was carried out according to the Ordinary Least Squares (OLS) analysis assumptions, which include normality, homoscedasticity, and endogeneity. The presence/absence of the omitted explanatory variable, which may limit the relevance of the model, was checked. Additionally, the presence or absence of the multicollinearity problem was also tested; it is a situation where it becomes difficult to identify the separate effect of independent variables on a dependent variable due to the existing strong relationships among them (Gujarati 2003). To test the existence of correlation between independent variables and to quantify the strength of their correlation, the Variance Inflation Factor (VIF) was used. As a rule, a VIF value greater than 10 indicates the variable is highly collinear; values greater than 3 show the presence of weak collinearity between the analyzed variables. Similarly, the existence of correlation between the selected dummy variables was tested using the Standard Contingency Coefficient (SCC) as described by Gujarati (2003). The correlation coefficient value ranges from +1 to −1, where +1 indicates perfect positive correlation, −1 indicates perfect negative correlation, and 0 indicates no correlation at all.

$$C=\sqrt{\frac{X^{2}}{N+X^{2}}}$$

where C = Stands for contingency coefficient, *X* ^2^ = is the chi‐square random variable, and *N* = is the total sample size of the study.

## 3 Results and Discussion

### 3.1 Demographic Character of the Respondents

The survey results indicate the majority of respondent households (89.8%) were male‐headed, while 10.2% were female‐headed households. This gender disparity in households may have its own implications for the gender‐specific activities, decision‐making power, and developing strategies, as reported in studies done on rural household dynamics (Farnworth et al. 2018). Concerning the camel production and management practiced, the large proportion of male‐headed households, as observed in the study area, related to gender‐specific activities, is less likely to be affected; because camel production is labour intensive and also major activities such as feeding, watering, treating sick animals, as well as decision power for selling live animals were male‐headed households.

Out of the total respondents interviewed, 62.1% were not able to read and write, whereas only 14% completed their primary and secondary school. Similar educational status was reported in different parts of the country: in the Afar region, 60% were illiterate (Yigzaw 2014); in the southern pastoral area, 51.3% (Gemechu et al. 2023); and in the Somali region, 79% (Wolkaro et al. 2017). The study results show that the proportion of respondents who did not attend formal education was higher; this may have a negative effect on milk production and marketing. As reported earlier, the academic status of the pastoralists had an implication for the utilization of extension services and new technology adoption. In fact, education helps the pastoralist to achieve the objective easily and increase the level of awareness. Kuma (2012) argues that literate persons easily accept the extension service delivered. It implies that the educational level of the respondent could be a barrier to the expansion of new interventions and extension services.

The average age of respondents was 49.12 ± 10.63 years, with a minimum of 25 and a maximum of 73 years. Out of the total respondents, half a percent of the respondents were aged < 48 years; this might be an opportunity for camel milk producers and productivity improvement. Moreover, this age distribution has implications for the knowledge and practices related to camel milk production and marketing. Older individuals possess valuable traditional knowledge but are often less flexible and less willing to accept new technology, while younger people might be more flexible and easily adopt new developmental technologies (Elias et al. 2015). A similar age distribution was reported by Gemechu et al. (2023) in the Borana and Guji zones, where the majority of the respondents were aged 30–50 years, and in the Somali region, the mean age was 47 years (Mahamud 2022).

The overall respondent household family size was 6.88 ± 2.17. The family size distribution had a positive implication for herd management. Camel production is labour‐intensive in nature, so the larger the family size, the more adventurous they are. However, large family sizes in the household require more milk; milk was the main source of food. In eastern Ethiopia, household family size showed a significant effect on daily milk utilization; large family sizes need larger amounts of milk for consumption, which reduces marketable surplus (Bekele et al. 2002). Consistent family size was reported by other scholars; Gemechu et al. (2023) reported in Borana, Guji, and West Guji that the average family size was 5.43; in the Afar region, it was 6.24 (Yigzaw 2014); and in the Somali region, it was 7.45 (Mahamud 2022).

Regarding the religion of the respondent, 52.3% of the respondents follow the Muslim religion, while 22.1% and 25.5% follow Protestant and Wakefata, respectively; the variation across the study district was significant (*p* < 0.005). In the Somali and Afar pastoral communities, camel milk was mainly consumed by Muslims (Gebremichael et al. 2019). The study finding indicates, unlike other camel producer communities like the Afar and Somali regions, as stated by the previous study, there is a wide religious heterogeneity in the study area. Such restriction of camel milk consumption by religious leaders significantly affects the amount of milk consumed or supplied for the market. This was an issue that inspired the researcher to focus on religious variation among respondent households as an explanatory variable to determine the amount of milk consumed or supplied to the market.

### 3.2 Camel Holding Size and Herd Composition

Table 2 shows summarized camel ownership per household in the study area; the overall mean value of camel holding size per head per household was 16.7 ± 5.88. The general linear model analysis result indicates camel ownership per household across the district was significantly (*p* < 0.001) different. The study result shows that even though the pastoralists in the study area started incorporating camels in their livestock composition lately, the camel population size per household has been increasing in the recent past. The camel ownership holding size observed in the study area was more or less similar to other camel milk producer areas, which is 14.69 and 12.34 in Gursum and Babile, respectively (Demissie et al. 2017); 19.88 ± 8.59 in the Somali region (Mahamud 2022); 19.6 ± 1.4 in the Boran zone (Megersa et al. 2008); and 12.2 in Guji and Boran zones (Gemechu et al. 2023). In the study areas, the number of camels owned per HH had a positive relationship with the social status and household food security. Particularly, as the number of lactating camels owned per household increases, the volume of milk produced also increases (Mahdi et al. 2025). Demissie et al. (2017) reported that in pastoral areas, surplus milk is supplied for a market.

**TABLE 2:** Herd composition and holding size of the respondent in the study area (*N* = 235).

Parameter	Camel herd composition per household by study district					*p*
	Dugda Dawa (*N* = 51)	Gomole (*N* = 80)	Dubluk (*N* = 56)	Moyale (*N* = 48)	Overall Mean ± SD	
< 1 year	2.76 ± 0.97^b^	3.27 ± 1.22^a^	3.36 ± 1.59^a^	2.56 ± 1.27^b^	3.04 ± 1.32	0.0021
> 1 < 4 year	6.94 ± 3.14^a^	6.05 ± 2.65^b^	3.71 ± 1.69^c^	4.02 ± 2.25^c^	5.27 ± 2.81	< 0.001
Lactating camel	3.68 ± 1.14^ab^	3.91 ± 1.4^a^	3.54 ± 1.4^ab^	3.20 ± 1.2^b^	3.63 ± 1.33	0.0318
Dry camel	1.25 ± 1.21^b^	3.19 ± 1.82^a^	2.95 ± 2.77^a^	2.70 ± 1.64^a^	2.61 ± 2.08	< 0.001
Adult male	2.0 ± 0.85^b^	2.83 ± 1.03^a^	1.27 ± 0.45^c^	2.17 ± 1.04^b^	2.14 ± 1.06	0.001
Total	16.68 ± 4.65	19.26 ± 6.34	14.58 ± 5.32	14.66 ± 5.32	16.7 ± 5.88	

*Note:* Different superscript letters in row indicate significant differences; Adult male means, camel aged > 4 years.

Abbreviation: *N* = the number of respondents.

The study result showed that out of the total number of camel population owned, 2714 (69.75%) were female and 1177 (30.25%) were male, implying that the higher proportion of females in the herd ensures the production potential of milk and provides a good opportunity for milk production. In comparison to the camel herd composition reported, it was in line with others: 74% in the Somali region (Wolkaro et al. 2017); 77.3% in the Afar region (Megersa et al. 2024); 68.84% at the national level (CSA 2022). Concerning the proportion of the camel population based on age, 1951 (50.14%) of the camels were aged 4 years and above. The present study's finding was lower than that of the CSA (2022) report, in which 72% of camels were 4 years and older. The variation observed in the study area might be associated with keeping camels. The overall mean of lactating camels owned per household in the study area was 3.63 ± 1.33, and across the district, it was significantly (*p* < 0.05) different. It was higher compared with the result of a study done in the Borana zone, 3.08 ± 0.34 (Wako 2015).

### 3.3 Milk Production Potential of Camel

Table 3 shows a summary of the milk production performance of camels in the area. In the study area, the overall mean value of milk produced per camel per day in liters was 5.92 ± 0.89 and 4.72 ± 0.83 during the wet and dry seasons, respectively, and across the district, the milk yield during the wet season was significantly (*p* < 0.005) different. The literature indicates the milk yield per camel per household per day reported in different areas varied: in Borana, 5.60 ± 2.50 and 3.54 ± 1.35 L during the wet and dry seasons, respectively (Legesse et al. 2023); in the Somali region, 4.90 ± 0.17 and 3.62 ± 0.134 L during the wet and dry seasons, respectively (Wolkaro et al. 2017); in the Mieso district, 7.12 ± 0.33 and 3.85 ± 0.203 L during the wet and dry seasons, respectively (Hussen et al. 2008); in the Somali region, 5.16 ± 0.15 L (Kebede et al. 2015); in the East Hararghe, 4.8 L (Demissie et al. 2017); in the Afar region, 4.2 L (Gebremichael et al. 2019). However, the study's findings resulted in higher figures compared with the national report; the average milk yield per day per camel is 2.54 L (CSA 2022). It indicates that the milk production potential of camels in the study area is relatively better; the amount of milk production variation reported might be associated with the availability of feed and water, herd management practices, breed variation, and lactation stage (Faraz et al. 2021). The study results were also indicative of camel milk production receiving particular recognition for its nutritional and income source for the pastoral community.

**TABLE 3:** Milk production performance of camels in the area (*N* = 235).

Milk yield per day per household during the wet and dry seasons by study District					
Variables	Dugda Dawa (*N* = 51)	Gomole (*N* = 80)	Dubluk (*N* = 56)	Moyale (*N* = 48)	*p*
Milk yield (L) wet season	5.84 ± 0.74^b^	6.18 ± 0.97^a^	5.64 ± 0.79^b^	5.87 ± 0.91^b^	0.0045
Milk yield (L) dry season	4.63 ± 0.97^ab^	4.88 ± 0.51^a^	4.51 ± 0.90^b^	4.77 ± 0.94^ab^	0.0646
Lactation length in months	11.88 ± 1.05^b^	13.01 ± 1.38^a^	12.67 ± 1.47^a^	12.87 ± 1.28^a^	< 0.001
Calving interval in months	30.50 ± 2.50^a^	27.06 ± 2.52^c^	28.69 ± 2.57^b^	28.72 ± 3.35^b^	< 0.001

*Note:* Different superscript letters in a row indicate significant differences.

Abbreviation: *N*, Number of respondents.

The survey revealed that, in the area, the average lactation length in months was 12.65 ± 1.37, and across the study district, it was significantly (*p* < 0.001) different. The result of this study was in line with others; in the south pastoral area, it was 12.86 months (Gemechu et al. 2023), and in the Somali region, Fafan zone, it was 13 months (Kebede et al. 2015). It was slightly lower than the lactation length reported in eastern Ethiopia, which was 10 months (Demissie et al. 2017), and 9 months at the national level (CSA 2022). In the area, the mean value of the calving interval was 28.54 ± 2.98 months, and across the study districts, it was significantly (*p* < 0.001) different. It was consistent with other study findings; in pastoral areas, the calving interval of camels ranged from 18 to 26 months (Yosef et al. 2015; Megersa et al. 2024). The calving interval result variation observed in the study area might be associated with the agro‐ecological condition of the area, breed difference, and level of management (El‐aziz et al. 2022).

### 3.4 Amount of Milk Produced, Consumed, and Sold in the Study Area

Table 4 shows the summarized amount of camel milk produced, consumed, and sold per HH per day. The overall mean for camel milk produced per HH per day was 13.2 ± 4.8 L during the wet season and 10.7 ± 4.75 L during the dry season. It indicates that the amount of milk produced in the study area is consistent with other camel producer areas. In the Somali region, 17.33 ± 0.58 and 15.10 ± 0.53 L were produced during the wet and dry seasons, respectively (Mohammed et al. 2016); in the Mieso district, 13.19 ± 0.95 and 7.63 ± 0.82 L were produced during the wet and dry seasons, respectively (Hussen et al. 2008). Milk yield variation observed in the study area might be due to breed differences, number of lactating camels, lactation stage, and feed and water availability. Hence, the availability of feed and water and the genetic makeup significantly influence the production and productivity of camels (Bekele et al. 2002; Megersa et al. 2008). The amount of milk produced per household has its own implication for the amount of milk supplied for market; as the amount of milk produced per household per day increases, the volume of milk market supplied also increases (Bekele et al. 2002; Noor et al. 2013).

**TABLE 4:** Amount of milk produced, consumed, sold, and social gifts in the area (*N* = 235).

Variable	Study district					*p*
	Parameter	Dugda Dawa (N=51)	Gomole (N=80)	Dubluk (N=56)	Moyale (N=48)	
Milk allocation during the wet season	Milk produced	11.1 ± 4.5^a^	16.4 ± 4.9^b^	12.6 ± 4.8^b^	12.7 ± 4.9^b^	0.0001
	Milk consumed	3.4 ± 1.3^b^	4.3 ± 1.6^a^	3.3 ± 1.4^b^	3.6 ± 1.5^b^	0.0001
	Milk sold	6.8 ± 2.6^a^	11.2 ± 3.6^c^	8.5 ± 3.1^b^	7.7 ± 3.1^bc^	0.0001
	Social gift and other	0.5 ± 0.3^b^	0.8 ± 0.4^a^	0.7 ± 0.4^a^	0.7 ± 0.4^ab^	0.002
Milk allocation during the dry season	Milk produced	9.2 ± 5.2^a^	12 ± 5.4^a^	10.3 ± 4.4^ab^	11.3 ± 4^b^	0.0154
	Milk consumed	2.7 ± 1.5^b^	4.5 ± 1.2^a^	3.1 ± 1.3^bc^	3.4 ± 1.5^c^	0.0001
	Milk sold	6.2 ± 3.7	7.1 ± 3.7	6.6 ± 3	6.0 ± 2.7	0.2629
	Social gift and other	1.4 ± 0.7^a^	0.9 ± 0.3^b^	0.5 ± 0.2^c^	0.3 ± 0.2^d^	0.0001

*Note:* Different superscript letters in rows indicate significant differences; No superscript letter for milk sold during dry season means no significant difference.

Abbreviation: *N*, the number of respondents.

The amount of milk consumed per day depends on the demand for milk, the season of the year, and the volume of milk produced. In the study area, the amount of milk consumed per HH per day during the wet season was 3.65 ± 1.45 L, whereas 3.43 ± 1.38 L was consumed during the dry season. The current study results indicated that around one‐third of the milk produced per HH was allocated for home consumption. Previously, similar milk consumption proportions were reported by other researchers; in the Somali region, 3.80 ± 1.01 L of milk produced was consumed (Mahamud 2022); in the Borana zone, on average, 3.7 ± 0.5 L was consumed (Megersa et al. 2008). The amount of milk consumed per household has a negative relationship with the volume of milk supplied for market (Noor et al. 2013).

In the study area, on average, 8.55 ± 3.1 and 6.48 ± 3.28 L of milk were supplied for the market during the wet and dry seasons, respectively. Across the district, the amount of milk supplied to the market during the wet season varied significantly (*p <* 0.001). The study finding was consistent with other scholar reports; in the Boran zone, 5.91 ± 0.38 and 4.12 ± 0.27 L were sold during the wet and dry seasons, respectively (Wako 2015); in the Afar region, 6.29 ± 0.11 and 3.64 ± 0.10 L of milk produced were sold during the wet and dry seasons, respectively (Mohammed et al. 2016). It was lower compared with the study reported in Afar, which was 14.3 L (Gebremichael et al. 2019).

The study result indicates that the amount of milk supplied for a market during wet and dry seasons varied; during the wet season, the amount of milk supplied for the market was higher; this variation might be associated with the amount of milk produced and the purpose of production. It was in line with a study done in the Somali region; the household demand of camel milk affects the amount of milk market supply negatively (Wako 2015). This idea was supported by other researchers; in the Afar region, during the dry season, the amount of milk sold decreased by 29.6% (Mohammed et al. 2016), and in the Mieso district, it decreased by 28% (Hussen et al. 2008).

The study result showed that the milk utilization proportion of the respondent depends on the amount of milk produced, household milk demand, and the purpose of production. The survey result showed that, out of the total amount of milk produced during the wet and dry seasons, around 1761.73 L (63.1%) were supplied to the market, and during the wet season, out of the total amount of milk produced, around 879.97 (28.77%), 2014.82 (65.86%), and 164.27 (5.37%) were allocated for home consumption, supplied to market, and used for social gifts/other purposes, respectively, and across the study district it was significantly (*p* < 0.001) different. Social gifts mean the milk allocated for the guests or relatives who have no milking animal to strengthen their social relationships. A similar milk utilization proportion was reported in Borana: 29% and 71% of the milk produced was allocated for home consumption and market supply, respectively (Wako 2015). In the Afar region, more than half of the milk produced was supplied to the market (Mohammed et al. 2016). In pastoral areas of Ethiopia, around 77.8% of the milk produced was supplied for market (Megersa et al. 2024). The variation observed might be associated with the amount of milk produced per household per day. Hence, the season can determine the availability of feed/water, which will affect the amount of milk produced and sold (Hussen et al. 2008).

### 3.5 Camel Milk Marketing System Practiced in the Study Area

#### 3.5.1 Milk Marketing and Market Outlet

The survey revealed that all 235 respondents (100%) interviewed participated in milk marketing. It agrees with a study done by Demissie et al. (2017); in the eastern part of Ethiopia, almost all camel milk producer households sold the milk. In the study area, only fresh camel milk was sold, and the consumers did not have any possibility to buy and consume processed milk and milk products. The majority (61.3%) of respondents sold the milk at urban markets, whereas 27.2% and 11.5% sold at the main roadside and farm gate, respectively. The milk marketing system significantly determines milk quality as well as its profitability. Hence, the milk market price at the main roadside and farm gate was lower than in the urban market; it might be due to the limited participation of market actors. The finding result was consistent with a previous study; in Afar, the majority (58.5%) of the pastoralists sold milk at the market center, the main roadside (23.2%), and 18.3% at the farm gate (Gebremichael et al. 2019). In the area, informal raw milk marketing was dominant, and it was carried out by females (75.7%) and adult females (24.3%). The study findings agree with the previous study done in the Afar and Somali regions; milk was mainly marketed informally, and entirely done by women (Amenu et al. 2024; Montgomery 2021; Mohammed et al. 2016). The milk market price was fixed by negotiation (42.3%) and demand and supply (28.4%). To meet the growing demand for camel milk and milk products, as well as to enhance milk marketing profit, the market infrastructure and awareness should be improved.

#### 3.5.2 Role of Gender in Dairy Sector in Pastoral Areas of Ethiopia

In the study area, across the market chain, milk marketing was mainly done by women. In addition to these, activities like milk handling and preservation practices at the household have been the main responsibility of women, which shows that activities related to milk marketing and handling have been entirely done by women. However, women's involvement in milk marketing was limited by different factors, such as traditional norms and limited resources. The study's findings were consistent with other studies; in most of the pastoral areas of Ethiopia, women have decision‐making power on milk and milk product marketing (Bekele et al. 2021; Demissie et al. 2017; Wolkaro et al. 2017). Interestingly, in the study area, camel milk production and marketing provide business opportunities that enhance women's empowerment and overall dairy productivity. To overcome those limiting factors and to empower women economically, organizing them into groups will provide a better platform for credit and market opportunities (Montgomery 2021).

#### 3.5.3 Milk Market Price

Table 5 shows the milk price analyzed during the study period (August 2024). The average raw milk price per liter in Ethiopian Birr (ETB) was 72.8, which was equivalent to 1.26 USD, and 89.8 ETB = 1.55 USD during the wet and dry seasons, respectively; across the district, it was significantly (*p* < 0.001) different.

**TABLE 5:** Mean comparison of camel milk market price in the study area (*N* = 235).

Variable	Study districts				*p*
	Dugda‐Dawa	Gomole	Dubluk	Moyale	
Milk price/L wet season	70 ± 4.6^b^	67.6 ± 6.3^c^	71.2 ± 5.8^b^	86.5 ± 4.4^a^	0.0001
Milk price/L dry season	92.2 ± 6^b^	83.7 ± 4.8^c^	85.4 ± 5.1^c^	102.7 ± 6.5^a^	0.0001

*Note:* Different superscript letters in row indicate significant differences; as the currency exchange rate during study period (August 2024): 1USD = 57.739 Ethiopian Birr.

Milk price was affected by the season of the year, production potential, and distance from urban towns, with a higher price during the dry season and closer to urban towns. Gomole district has a production potential, but due to poor market availability, the milk market price per liter was lower, 67.6 ± 6.3 and 83.7 ± 4.8 in ETB during the wet and dry seasons, respectively. On the other hand, the milk market price in ETB was higher in the Moyale district, 86.5 ± 4.4 and 102.7 ± 6.5 during the wet and dry seasons, respectively. According to Demissie et al. (2017) study findings, the milk price is one of the driving factors that determine the amount of milk supplied for a market. The study findings indicated that the milk market price varied across the study districts; it might be associated with the market availability.

#### 3.5.4 Camel Milk Market Channel

**FIGURE 3:**
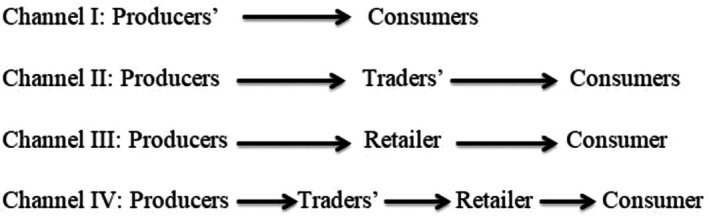
Flow chart of camel market channel identified in the study area.

In the study areas, four major camel milk market channels were identified and the milk market flow chart is presented in Figure 3. Across the market chain, the amount of milk passed through each milk market channel was significantly different. In the first milk market channel, the consumers directly purchase from producers, which accounts for 28.9% of the total, while the large volume (45.9%) of the milk was channeled through the second market channel, and around 71.1% of the producers sold the milk to traders. The study's findings resulted in lower than expected compared with others; in the south pastoral area, the majority (83.13%) of milk producers sold to traders (Gemechu et al. 2023). Traders collect the milk from producers and transport it to urban areas on a daily basis. From the total milk transported to the terminal market (Moyale), around 41.23% was marketed at the border of Ethiopia and Kenya, and the remaining 58.77% was sold in urban, peri‐urban, and rural areas. It is in line with Megersa et al. (2024); Coppock et al. (2005) in southern pastoral areas, a larger proportion of milk was marketed in neighboring countries like Kenya through Moyale Town. The third milk market channel accounts for 15.5% of the total amount of milk marketed; in this channel, the retailers purchase milk from producers (63.4%) and traders (36.6%); they sell it to restaurants, teashops, and individual consumers. The current study findings are in line with a study done in Afar; in the producer‐to‐retailer‐to‐consumer milk market channel, it accounts for 17.2% (Mohammed et al. 2016). The volume of milk passing through the fourth market channel was lowest (9.7%). In all milk market channels, transporters play a great role in transporting milk from production areas to urban centers (market centers).

### 3.6 Milk Market Actors

#### 3.6.1 Traders

The socioeconomic characteristics of the traders interviewed indicate that all 92 respondents (100%) were females. It agrees with the Bekele et al. (2021) report that in the Borana zone, the majority of milk traders were women. Regarding educational status, the majority (> 84%) of the traders interviewed were not able to read and write, while 15.23% had formal education; this was in line with Wolkaro et al. (2017), in the Somali region, 88% of traders were not able to read and write. The higher proportion of illiteracy observed in this study has its own effect on new technology adoption and business management. The study result indicated that respondents' educational status clearly reflects the need for further improvement in their educational status. Concerning their age, the majority (55.46%) of traders were aged 28–45 years, while 21.74% were aged 18–27 years old, and the rest, 22.8%, were over 45 years old. The average family size was 8.3 ± 1.01; the experience they had in milk marketing ranged from 4 to 18 years. It was in line with Wolkaro et al. (2017); in the Somali region, the average family size of the traders was 8.5. Around 41.3% of traders participate only in camel milk marketing, while 58.7% are engaged in camel and cow milk marketing. A similar trader engagement distribution proportion was reported in East Hararghe, where 49% of traders participate only in camel milk marketing (Demissie et al. 2017).

#### 3.6.2 Consumers

Regarding consumer households interviewed, out of the total respondents interviewed, 67.3% were male, and the remaining 32.7% were female. The educational status indicates that around 37.4% were not able to read and write, while 63.6% had attended formal education. Unlike producer households, the majority of consumers attended formal education, and the higher level of education observed among consumers might be associated with the expansion of education in urban areas. Regarding the respondent age group, the majority (46.7%) was aged between 30 and 44 years, while 30.8% and 22.4% were aged 45–60 and < 30 years old, respectively. The presence of a higher proportion of individuals aged between 30 and 44 years and educated persons implied an increasing awareness of the benefits of camel milk and improved milk handling strategies in the areas. Hence, facilitation of market‐based awareness for households is recommended as a means of enhancing consumer demand for quality milk (Ait El Alia et al. 2025).

The respondent's experience in consuming milk indicates that around 41.1% of the respondents had 1–5 years, while 32.7% and 26.2% had between 5 and 15 years and more than 15 years, respectively. The average amount of milk consumed per household per day was 0.54 ± 0.37 L. The majority (42.1%) of the respondent households consume less than one cup of milk per day. A recent study finding also indicated that the majority of consumers drink less than one cup of camel milk per day (Ait El Alia et al. 2025). The source of milk for consumer households was from producers (43.9%), traders (36.4%), and retailers (19.6%). This idea was supported by another scholar, named Montgomery (2021), who found that in pastoral areas, the majority of camel milk consumers purchase milk from producers' households, as it is more affordable and convenient.

**FIGURE 4:**
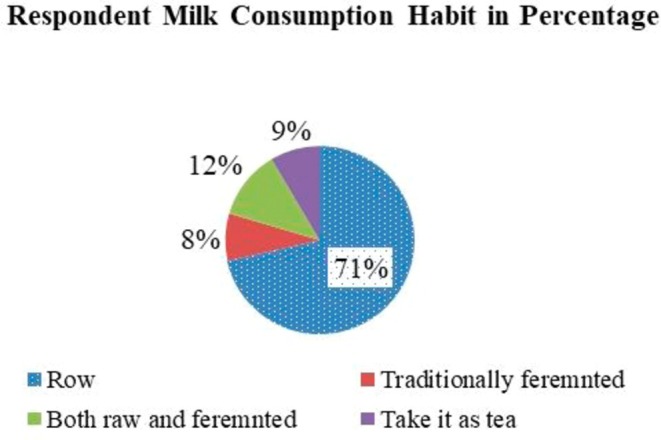
Indicates respondents' milk consumption habit; *Source:* Own survey result, 2025.

Respondent milk consumption habits revealed that the majority (71%) of the respondents consumed milk as its raw, whereas 8% consumed traditionally fermented, also known as Chuche (Figure 4). The present study results revealed that a significant proportion of the respondents consumed camel milk without any heat treatment. Indeed, the consumption of raw milk without any processing techniques has its own effect on the consumer's health. The consumption of raw milk observed in the study area might be associated with consumer perception; the majority (81.3%) of the respondents believe the consumption of raw camel milk has no drawbacks on their health. Additionally, inadequate awareness (12.2%) of the health risks of raw milk consumption was the other issue identified. The present study's findings are in line with others; the majority of the pastoralists didn't attend food hygiene training (Bekele et al. 2021). Similarly, the other authors stated that, in the Somali and Afar regions, the majority of pastoralists believed that raw camel milk was fresher, boosts the immune system, and contains higher nutrient levels (Noor et al. 2013; Mahamud 2022). Therefore, the governmental/nongovernmental organization should work on awareness creation for consumers on public health risks.

#### 3.6.3 Transporters

Concerning transporters interviewed, all 47 (100%) were male. In the study area, producers and traders transported milk from rural areas to urban market centers using transportation such as motorbikes, Isuzu/pickup trucks, women's backs, and draught animals. Observations made during the field survey confirmed that milk was transported to market using two segments. The first segment was transporting milk produced from the production area or pastoral homestead to the roadside or collection centers. It was mainly carried out using motorbikes and women's backs. In the second milk transportation method, the milk was transported from collection centers or roadsides to urban areas; it was usually delivered using public transport. Transporters stated that the traders and producers were not usually traveling during milk transportation; they sent it by drivers, putting unique marks on the milk‐handling containers. In the area, milk was transported using 10‐ and 20‐L plastic *Jerry‐cans*. The transportation cost paid was based on the amount of milk transported and the distance traveled. During the study period, the average transportation cost paid to transport 20 L of milk from Gomole district to Moyale district (245 km) was 100 ETB, with an average cost of 4.7 ETB per liter. Whereas, to transport 20 L of fresh milk from the Dugda‐Dawa district to Moyale, which needs to travel 270 km, was 110 ETB, with an average cost of 5.3 ETB paid per liter during the study period.

### 3.7 Services Providers

#### 3.7.1 Veterinary Service

The present study result showed that only one‐third of the respondent households had access to veterinary services. Due to inadequate veterinary service, 57% of the respondents treat sick animals by themselves. The vaccination coverage was lower (31.6%); in line with a study done in the Somali region, 33.5% of the respondents vaccinated their camel (Igge et al. 2020). The veterinary service access has a great impact on camel milk production and milk marketing because it affects milk production and productivity. Diseases such as mastitis and parasites can affect the production and productivity of dairy animals negatively, resulting in economic losses (Mahdi et al. 2025). A consistent study result was reported in other pastoral areas; in the Somali region, veterinary service access was inadequate (Bekele et al. 2002; Temesgen et al. 2024). The study results indicated that the veterinary services access needs further intervention. Therefore, to enhance dairy productivity, the government should improve veterinary services through awareness creation and provision of vaccination and/or treatment.

#### 3.7.2 Market Information

Information is the driving factor for the market, and it has a positive impact on milk production and marketing. In the study area, the market information access of the respondents was only 37.4%. However, access to market information has a positive effect on market participation and the quantity of milk supplied for the market (Wolkaro et al. 2017). The study result is in line with the study done in the Somali region; 36.5% of respondents had access to market information (Wolkaro et al. 2017). Relative pastoralists (39.15%) and traders (31.9%) were the sources of market information used, whereas mobile phone and extension agents accounted for 20.85% and 8.01%, respectively. According to Igge et al. (2020) report, in eastern Ethiopia, as an information source, the number of respondents who get market information from their relative pastoralist was higher (37.5%), next to mobile phone (14%), and traders (15.5%). As reported in other pastoral areas of Ethiopia, pastoralists were the main source of market information (Farnworth et al. 2018). The study result indicates that the number of respondents using a mobile phone as a milk market information source is limited. Therefore, to improve market information, governmental/nongovernmental organizations should promote a cost‐effective and easily manageable information system.

### 3.8 Factors Affecting Milk Market Supply in the Study Area

#### 3.8.1 Regression Model Analysis Result

Table 6 shows the summarized Ordinary Least Squares model results. Before fitting the variable, the post‐estimation diagnosis assumption was tested. Normality and model specification test: the analyzed result indicates the selected model is fit to analyze the linearity of the selected variable (*p* < 0.001). The adjusted *R* ^2^ value was 0.8544; it implies that around 86% of the variation in milk market supply observed in this study was due to the selected exploratory variables, while other factors that are not included in this research contribute around 14% to milk market supply variations. The overall mean of VIF was 2.04; this indicates the absence of a multicollinearity problem (weak correlation) between selected variables.

**TABLE 6:** Ordinary least squares (OLS) model analysis results.

Variables	Coefficients	Robust standard error	*t* ratio	*P>/t/*
Family size	−1.165503	0.3277663	−3.56	0.0001^a^
Religion	−4.132653	1.273641	−3.24	0.001^a^
Educational status	6.332944	1.84859	3.43	0.001^a^
Other income source	0.2602393	1.283157	0.20	0.839
Amount of milk produced (L)	0.7713784	0.0490801	15.72	0.001^a^
Amount of milk consumed (L)	−0.6799256	0.1451314	−4.68	0.001^a^
Market distance (km)	−0.1782687	0.0534927	−3.33	0.001^a^
Milk price (ETB)	0.3573756	0.1119594	3.19	0.002
Market information access	7.1153	1.476841	4.82	0.0001^a^
Number of lactating camels	0.0508343	0.9009988	0.06	0.955
Veterinary service	−1.283923	1.392516	−0.92	0.358
Constant	−3.443128	8.951367	−0.38	0.701

*Note:* Dependent variable: amount of milk supplied for a market; Adjusted *R* ^2^ = 0.8544.

^a^ Indicates a regression relationship is significant at 1%.

Further, the presence or absence of an endogeneity problem among variable it was tested using the *Two Stage Least Squares* (*2SLS*) model; it was analyzed based on their relationship with the amount of milk produced as the instrumented variable and access to veterinary service as the instrumental variable, and the analyzed model results showed it has no endogeneity problem (*p* > 0.05). The robust regression model results show that total family size, the amount of milk produced, religion, the amount of milk consumed, education status, milk price, and market information significantly (*p* < 0.001) determine the amount of milk market supply; however, other income sources, the number of lactating camels, and veterinary services did not significantly contribute to the amount of milk market supply.

The regression model result shows that the total family size, as expected, had a negative relationship with the amount of milk supplied for market (*p* < 0.001). It indicates that when the family size increases by one person, the amount of milk market supply declines by 1.2 L; hence, in most of the pastoral areas, household milk was used as the main source of food. Furthermore, the religion of the respondent determines the amount of milk supply significantly (*p* < 0.001). When we compare the Muslim religion followers with others like Protestant and Orthodox, the amount of milk market supplied by Muslims is decreased by 4.13 L.

Educational status of the respondent was hypothesized, and the regression model result showed that it had a significant (*p* < 0.001) effect on the amount of milk market supply positively. When we compare a non‐educated person with an educated one, the amount of milk market supplied by the non‐educated person is decreased by 6.3 L. As expected, the amount of milk produced per household significantly (*p* < 0.001) determines the volume of milk sold positively. The model result indicates that when milk production increases by 1 L, it also increases the amount of milk market supply by 0.77 L, indicating that the more they produce, the more they supply for the market. The amount of milk consumed per household significantly (*p* < 0.001) determines the amount of milk supplied for a market negatively. As expected, the model results also indicated that when the amount of milk consumed increases by 1 L, the amount of milk market supply may decrease by 0.68 L.

The model result depicted that market distance traveled to reach the market center, as expected, had a negative relationship with the quantity of milk supplied for a market (*p* < 0.001). It indicates that when the market distance traveled increases by 1 km, the amount of milk sold declines by 0.19 L; it implies that when the market is more accessible for pastoralists, the amount of milk market supply also increases. The model result depicted that the milk price has a positive relationship with the amount of milk market supply, and it was significant (*p* < 0.001). The result showed that when the price of milk increases by one Eth. Birr, the amount of milk market supply also increases by 0.36 L; it implies that when the price of milk in the market increases, the pastoralist supplies more milk for the market. As hypothesized, the access to market information had significantly (*p* < 0.001) determined the amount of milk market supply positively; the model result indicates, as compared with those having market information access, having no market information access decreases the amount of milk market supply by 7.11 L, suggesting that market information access for the pastoralist had a great role in improving the amount of milk market supply.

### 3.9 Challenges and Opportunities of Milk Production and Marketing

Summaries of challenges identified related to producers and traders in the study area were presented in Table 7. Accordingly, feed and water shortage (0.30), disease and parasites (0.28), market inaccessibility (0.19), lack of extension service (0.10), and lack of policy support/input supply (0.14) were the challenges identified related to producers. Other empirical studies also highlighted that feed and water shortage, poor market infrastructure, insufficient veterinary services, market distance, and inadequate extension service were the top constraints producers reported in eastern areas of Ethiopia (Igge et al. 2020; Megersa et al. 2024; Hussen et al. 2008).

**TABLE 7:** Camel milk producers and traders' challenges identified in the study area.

The major challenge of milk producers' households and traders identified in the study area					
Milk producers challenge	Index	Rank	Milk traders challenge	Index	Rank
Disease and parasite	0.28	2nd	Poor transportation facility	0.31	1st
Feed and water scarcity	0.30	1st	Inadequate market infrastructure	0.28	2nd
Market accessibility	0.19	3rd	Seasonal availability of milk	0.14	4th
Policy support/input supply	0.14	4th	Inadequate capital and skill gap	0.07	5th
Lack of extension service	0.10	5th	Lack of market linkage	0.20	3rd

*Source:* Own survey result, 2025.

The primary factors limiting the growth of dairy in pastoral areas, according to OPaDCC (2021), are a lack of regulations that are unresponsive to the demands of the pastoral systems. If not adequately handled, the market infrastructure and production‐related factors such as feed scarcity, disease prevalence, and lower producer awareness have a detrimental impact on milk production and marketing. By increasing the amount of milk produced per household and market supply, the improvements we implemented with an emphasis on veterinary care, market access, and input will enhance the revenue from milk marketing (Gemechu et al. 2023). An improvement in market access for producers is necessary to supply more milk; the nearest market encourages the producers to sell more milk (Temesgen et al. 2024). The study's findings show that the constraints identified in the study area are interrelated; hence, a comprehensive strategy is advised to address them. Additionally, the government should pay attention to and focus on improving the infrastructure, especially the market, veterinary care, and feed and water availability.

Regarding the challenges identified related to traders, poor transportation (0.31), inadequate market infrastructure (0.28), and lack of milk market linkage (0.20) were the top‐ranked traders' problems. Furthermore, seasonal variation in the availability of milk, lack of capital, and inadequate business management skills were also challenges mentioned. Challenges highlighted in the study area were in line with the study results found by other authors: poor transportation facilities, inadequate milk market infrastructure, seasonal availability of milk, and lack of market linkage were the main challenges mentioned by traders (Gemechu et al. 2023; Igge et al. 2020; Bekele et al. 2021). Hence, further intervention is needed to overcome the traders' challenges, especially those associated with market infrastructure and financial service problems. Facilitation of market linkages and creating awareness of basic business management skills through extension services will help traders access better prices for their products.

In the study area for the last 2 years, the camel population has been increasing, associated with climate variability, vegetation cover change, feed, and water scarcity. The idea was supported: the camel population in Ethiopia has grown rapidly in the last few decades, at a rate of 4.5% per year (Megersa et al. 2024). The traditional knowledge of the pastoralist on how they kept a large proportion of females in their herd can have a great contribution to milk production; it also provides opportunity for milk producers and marketing. Moreover, due to rapid urbanization, over time the demand for camel milk at the local market has been increasing. This idea was supported by another study in the Somali and Afar regions, where the demand for camel milk at local and export levels had markedly increased over the last decades (Muloi et al. 2018; Mahamud 2022).

### 3.10 Limitations and Future Research Direction

This study provides useful insights into camel milk production potential, the market chain, and highlights key areas that need further investigation. The scope of the study was geographically limited to the southern pastoral areas of Ethiopia, which might affect the generalization of the research findings to support decision making at the country level. Also, it is limited in analyzing the market chain aspects, including milk production, market channel, milk price dynamics, market chain actor identification challenges, and opportunities. Therefore, to improve milk marketing sustainably and deepen the understanding of other important value chain components, such as value addition, market margin, cost‐benefit analysis, and the role of governance, all need further investigation.

## 4 Conclusion and Recommendation

In this study, researchers attempted to analyze the camel milk market chain and identified key factors influencing market supply in the Borana and West Guji zones of southern Ethiopia. In the study area, camel production and milk marketing play a significant role for the pastoral community. Camel milk marketing practiced was informal, and it was mainly carried out by women. From the four market channels identified, the producers to traders to consumers' milk channel was the most prominent, accounting for around 45.9% of marketed milk. Among the 11 explanatory variables analyzed, income sources, number of lactating camels, and access to veterinary services were not statistically significant in influencing supply volume.

Despite favorable conditions such as adaptable camel breeds, urbanization, and rising demand, several challenges persist, including: limited veterinary services, feed and water shortages, poor market infrastructure, weak extension services, and gaps in business knowledge. The findings of the study have its own implications for the international community in general and the study area in particular for promoting sustainable camel dairy production and marketing. In addition, to achieve the global milk and milk product demand and to maintain the sustainability of the dairy development sector, information that indicates the status of milk production, marketing systems practiced, and possible opportunities is critical.

### 4.1 Recommendations

To enhance milk yield and market efficiency sustainability, coordinated efforts by the government and NGOs are essential. Key interventions include:

- Rangeland management and development of water points
- Improved veterinary services, including vaccination and disease awareness creation
- Strengthening market infrastructure and linkages
- Capacity building for stakeholders through targeted training on milk marketing and quality

These measures will support sustainable dairy development and improve livelihoods in pastoral communities.

## Author Contributions

**Hussen Abduku:** conceptualization (equal), data curation (equal), formal analysis (equal), investigation (equal), writing – original draft (equal), writing – review and editing (equal). **Mitiku Eshetu:** conceptualization (equal), methodology (equal), supervision (equal), validation (equal), writing – review and editing (equal). **Takele Wolkaro:** conceptualization (equal), methodology (equal), supervision (equal), validation (equal), writing – review and editing (equal). **Tesfemariam Berhe:** conceptualization (equal), methodology (equal), supervision (equal), validation (equal), writing – review and editing (equal). **Temesgen Jembere:** conceptualization (equal), methodology (equal), supervision (equal), validation (equal), writing – review and editing (equal).

## Ethics Statement

In this study, the ethical approval statement was obtained from Haramaya University, School of Animal and Range Sciences Committee ID: SARS/92/2025. Additionally, prior to the interview, verbal consent was obtained from the respondent on their willingness to participate in the study. The respondents were also fully informed about the purposes of the research, confidentiality of responses during data analysis, write‐up, and publication.

## Conflicts of Interest

The authors declare no conflicts of interest.

## Data Availability

The dataset analyzed/generated during the study was obtained from the authors following the legal authorization.
